# Characterization of metabolic changes and adaptive remodeling during a hypobaric hypoxic exploration atmosphere environment

**DOI:** 10.3389/fphys.2026.1839091

**Published:** 2026-09-08

**Authors:** T. Fincke, S. M. Smith, B. Crucian, G. L. Douglas, P. Estep, A. Garbino, P. Gillman, M. Hew, T. Oswald, S. Rapley, M. Young, S. R. Zwart

**Affiliations:** 1 Texas Tech University, Lubbock, TX, United States; 2 Human Health and Performance Directorate, NASA Johnson Space Center, Houston, TX, United States; 3 Human Health and Performance Directorate, GeoControl Systems, Inc, Houston, TX, United States; 4 Human Health and Performance Directorate, JES Tech, Houston, TX, United States; 5 Human Health and Performance Directorate, KBR, Houston, TX, United States; 6 Human Health and Performance Directorate, University of Houston, Houston, TX, United States

**Keywords:** hyperoxia, hypoxia, iron, metabolism, spaceflight

## Abstract

**Introduction:**

The International Space Station maintains normoxic atmospheric conditions of 14.7 psia (101 kPa) and 21% oxygen. Astronauts don spacesuits to conduct extravehicular activities (EVAs), which operate at a reduced pressure of 4.3 psia (29.6 kPa), and a hyperoxic atmosphere of 100% oxygen. The reduction in pressure presents a risk of decompression sickness and is mitigated by a several hour denitrogenation (aka, prebreathe) protocol. To reduce the time required for prebreathe, future lunar and planetary exploration habitats and vehicles are expected to maintain a hypobaric, mildly hypoxic habitable environment in order to facilitate faster transitions to hypobaric hyperoxic EVAs. Among other things, hypoxic environments can influence oxidative stress and alter glucose metabolism, or other adaptive remodeling.

**Methods:**

Three 11-day chamber study missions (n=8/mission) were conducted to characterize the effects of alternating a mildly hypoxic living environment [8.2-9.6 psia (56.5 – 66.2 kPa)/28.5-34% oxygen] with hyperoxic EVAs [4.3 psia (29.6 kPa)/85-95% oxygen]. Fasting blood and 24-h urine samples were collected before the mission, and then again before and after EVAs on mission days (MD) 3 and 7, and a final collection on MD10.

**Results:**

Significant fluctuations of iron, amino acid, and glucose metabolism, along with changes in adaptive responses including some oxidative stress were observed before and after hyperoxic EVA simulations.

**Discussion:**

Based on these findings, it is evident that the effects of alternating hypoxia and hyperoxia may alter metabolism and physiology during exploration-class space missions.

## Introduction

The International Space Station (ISS) provides a normoxic environment, with pressure and oxygen concentration mirroring those at sea level on Earth (i.e., 14.7 psia/21% O_2_). ISS crews generally participate in Extravehicular Activities (EVAs) a few times over the course of a 6-month mission. The spacesuits used during EVAs operate at approximately 4.3 psia and 100% O_2_ (Abercromby et al., 2015). The transition between the higher pressure ISS cabin and the lower pressure EVA suit presents a risk of decompression sickness (DCS). To mitigate this risk, ISS EVA denitrogenation protocols are conducted by breathing 100% oxygen prior to depressurization (“prebreathe”). These protocols currently require a significant amount of crew time, on the order of 4 hours per EVA. While this overhead is acceptable for infrequent EVAs, future exploration missions to lunar and planetary surfaces are anticipating that astronauts will participate in EVAs 3–5 times a week, which will make this untenable; however, prebreathe duration can be reduced if the habitat’s operating environment is lower pressure with higher oxygen content, effectively reducing the nitrogen content in the body and thus reducing risk of DCS.

Long-term exposure to varied atmospheric pressure can lead to numerous physiological adaptive changes. Few studies of humans experiencing alternating hypoxic and hyperoxic atmospheres have been conducted; however, evidence exists that these can lead to metabolic changes, changes in iron homeostasis, including alterations of circulating metabolites, vitamins, oxidative stress, inflammation, and appetite (Choe et al., 2016; Karl et al., 2018).

Changes in body mass and physiology observed during Mercury spaceflights correlated with time spent in the Mercury full pressure space suit, and body mass loss was associated with changes in heart rate (Carpentier et al., 2018). Hypoxia exposure can shift fuel homeostasis to favor glycolysis and anaerobic metabolism over the more efficient oxidative phosphorylation (Murray, 2009; Levett et al., 2012; Murray, 2016). Alternating hypoxic and hyperoxic conditions routinely during a deep-space mission related to frequent EVA cadence may contribute to changes in caloric requirements, body mass and metabolism. Food provision on exploration-class missions represents a significant logistical challenge, and accurately defining caloric requirements will be critical for mission success.

To understand how multiple hypoxic/hyperoxic atmospheric transitions affect the human body, three 11-day chamber studies were conducted at the Johnson Space Center. Based on physiological adaptive changes to hypoxic environments in general, this study characterized similar endpoints related to oxidative stress, energy metabolism, iron status, bone resorption, and body weight.

## Materials and methods

### Subjects

24 subjects were recruited to participate in one of three 11-d missions in 2022-2023. Four of the subjects completed 2 of the missions, one for each ambient atmosphere, so there were a total of 20 unique individuals recruited (10 M/10 F, 36 ± 8 y) across these three missions. Subjects completed a modified U.S. Air Force Class 3 physical exam and provided written informed consent. Subjects weighed 76 ± 12 kg and were 175 ± 8 cm tall (BMI: 24.6 ± 2.4 kg/cm^2^). Each of the 11-d missions had 8 subjects: 6 of which participated in all simulated EVA activities while 2 subjects acted as ‘Doppler technicians’ (DTs) who did not participate in simulated EVA activities but instead served as in-chamber test operators. The DTs still experienced all ‘chamber variables’ including isolation, diet, circadian misalignment, and atmosphere cycles; however, the DTs were afforded more oxygen and prebreathe time as they were tasked with performing doppler and ultrasound data collection to assess decompression sickness risk along with collection of other supporting data.

Subjects consumed space foods during the mission and were provided with a menu that met their caloric requirements using the dietary reference intake predictive energy equations for active, normal weight adults (National Academies of Sciences Engineering and Medicine, 2023). Additional calorie requirements were determined to accommodate test EVA operations based on VO2 max outputs. Before the mission, subjects consumed their nominal diet and were not restricted in any way in terms of diet or supplement use.

### Environmental chamber and atmosphere

The three 11-day missions were conducted in a three-story, 20-foot diameter hypobaric chamber at the NASA Johnson Space Center (Anchondo et al., 2024). The nominal ambient atmosphere was 8.2 psia (56.5 kPa)/34%O_2_ for missions 1 and 2 and 9.6 psia (66.2 kPa)/28.5%O_2_ for mission 3 (**Table 1**). Chamber pressure and oxygen was controlled via vacuum pumps, compressed gas supplies and systems external to the chamber. Subjects completed an initial 3hr prebreathe at sea level to expedite denitrogenation and prevent any initial decompression stress. The chamber was then depressurized to the 8.2psia/34% O_2_ (missions 1 and 2) or 9.6psia/28.5% O_2_ (mission 3) atmosphere for subjects to adapt for 48 hours prior to conducting any EVA activities.

**Table 1:** Experimental design. 24-h urine collections were done before and on MD3, MD7, and MD10.

	Mission day											
	Pre (normoxic)	MD1 (hypoxic)	MD2 (hypoxic)	MD3 (hyperoxic EVA)	MD4 (hypoxic)	MD5 (hyperoxic EVA)	MD6 (hypoxic)	MD7 (hyperoxic EVA)	MD8 (hypoxic)	MD9 (hyperoxic EVA)	MD10 (hypoxic)	MD11 (hyperoxic EVA)
Psia	**14.7**	**8.2** *(9.6)*	**8.2** *(9.6)*	**4.3** *(4.3)*	**8.2** *(9.6)*	**4.3** *(4.3)*	**8.2** *(9.6)*	**4.3** *(4.3)*	**8.2** *(9.6)*	**4.3** *(4.3)*	**8.2** *(9.6)*	**4.3** *(4.3)*
%O2	**21**	**34** *(28.5)*	**34** *(28.5)*	**85** *(95)*	**34** *(28.5)*	**85** *(95)*	**34** *(28.5)*	**85** *(95)*	**34** *(28.5)*	**85** *(95)*	**34** *(28.5)*	**85** *(95)*
**24 h urine**	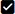			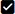				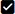			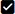	
**Fasted blood**	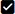			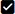 Pre/post EVA				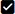 Pre/post EVA			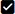	
**Body weight**	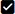	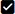	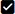	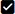	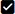	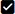	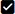	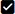	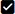	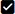	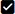	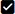

Mission 3 conditions in italics. Numbers in bold represent Missions 1 and 2.

Fasted blood draws were collected before the mission, and then before and after EVAs on MD3 and MD7, and in the morning before the EVA on MD10.

In the study, “hypoxic” refers to the reduced-pressure habitat environment used during non-EVA days (8.2-9.6 psia with FiO2 of 28.5-34%), which yielded inspired piO2 values around ~128 mmHg – lower than sea-level conditions but above classical clinical definitions of hypoxemia. Conversely, “hyperoxia” refers to the EVA phases conducted at 4.3 psia with 85-95% O2, resulting in inspired PO2 values of ~149–175 mmHg.

### Simulated EVAs

Following the equilibration period, subjects performed EVAs on alternating days (i.e. mission days, MD3, MD5, MD7, MD9, and MD11) of each mission. To simulate hypobaric hyperoxic EVAs, the chamber was depressurized to 4.3 psia for 6 hours. Subjects donned breathing masks (SOLR Oxygen Mask, Airborne Systems, Pennsauken, NJ) during the simulated EVAs. Subjects received 85% O_2_ for EVAs conducted during missions 1 and 2, and 95% O_2_ for EVAs conducted during mission 3. Prebreathe protocols were established to reduce risk of DCS. Missions 1 and 2 completed a 20min prebreathe prior to depressurization to EVA pressure (4.3 psia), while mission 3 included a 150min prebreathe prior to depressurization. During the hyperoxic EVAs, participants performed controlled exercise and fine motor skill tasks to simulate typical EVA activities. Tasks were switched every 5 minutes for a total of 6 h, with average workloads equivalent to 1200 BTU/h (~352 W) (Dunn et al., 2022; Garbino et al., 2024).

### Blood collection and biochemical analyses

Blood samples were collected once 5–7 days before each mission, before and after the simulated EVAs on MD3 and MD7, and once in the morning on MD10. Except for post-EVA collections, samples were collected fasting. The blood tubes were collected inside the chamber by a trained phlebotomist and transferred outside the chamber within 15 min of collection through a small air lock (“Transfer Lock”). The blood was allowed to clot for 30 min and tubes were then centrifuged to separate the serum.

Glucose and amino acid metabolites were measured by GC/MS or liquid chromatography/tandem mass spectrometry (LC/MS/MS) at Bevital (Bergen, Norway). Because of budget constraints, glucose and amino acid metabolites were only measured on fasting samples. Catalase was measured using a commercially available ELISA (Cayman Chemical, Ann Arbor, MI). Superoxide dismutase, total antioxidant capacity, total lipid peroxides, reduced and oxidized glutathione, glucose, insulin, c-reactive protein and oxidized LDL were measured as previously described (Lee et al., 2020; Douglas et al., 2022). HIF-1α and glial fibrillary acidic protein (GFAP) (Abcam, Waltham, MA), HSP70 (Invitrogen, Carlsbad, CA), and N-telopeptide (Zeus Scientific, Branchburg, New Jersey) were measured using commercially available ELISA kits. Serum and red blood cell folate were measured using a microbiological assay (Pfeiffer et al., 2016). Erythropoietin, ferritin, transferrin, total iron binding capacity (TIBC), and serum iron were analyzed using a clinical chemistry analyzer (Vitros Chemistry Analyzer 5600, QuidelOrtho, San Diego, CA).

### Urine collection and analyses

24-h urine samples were collected once before and on MD3, MD7, MD10. Urine was analyzed for n-telopeptide using a commercially available ELISA (Abbott Laboratories, Abbott Park, IL). Urinary creatinine was analyzed using a Vitros 5600 autoanalyzer (Ortho Clinical Diagnostics, Raritan, NJ). 8-hydroxydeoxyguanosine (8-OHdG) was determined using LC/MS/MS techniques (Waters Quattro, Waters Inc, Milford, MA) (Hosozumi et al., 2012).

### Body weight

Subjects weighed themselves daily each morning before and during the mission (Health o Meter HHM498L Professional Remote Digital Scale).

### Statistical analyses

A similar statistical approach to that used by Crucian et al. (2025) was applied in the present analyses. All models were fit in SAS v9.4 using the GLIMMIX procedure. For continuous measures, linear mixed-effects models included day/time (BDC AM; MD3 AM/PM; MD7 AM/PM; MD10 AM) as a categorical fixed effect. A random intercept for subjects nested within missions accounted for (1) repeated measurements within individuals, (2) subjects who participated in more than one mission, and (3) mission-level dependencies arising from shared operational conditions. Variance-components covariance structures were used, and degrees of freedom were estimated using the Kenward–Roger method.

Because missions differed in atmospheric pressure, oxygen concentration, and prebreathe procedures – but mission effects were not hypotheses of interest –mission-level variability was modeled as a random grouping factor rather than a fixed effect to account for inter-mission heterogeneity while avoiding over parameterization. With only three missions, treating mission as fixed would overparameterize the model, reduce power, and inefficiently allocate degrees of freedom. Nesting subjects within provided appropriate partial pooling and mitigated risk of inflated Type I error.

Amino acid and glucose metabolites were measured only at BDC AM, MD3 AM, MD7 AM, and MD10 AM. Because PM (post-EVA) measurements were not available for these measures, analyses were limited to hypoxia-period AM timepoints, and AM–PM contrasts were not performed. For biomarkers with measurements at both AM and PM on MD3 and MD, planned contrasts evaluated post-EVA (PM) vs pre-EVA (AM) on the same day.

Estimated marginal means (EMMs) with 95% confidence intervals were reported. EMMs were used to estimate changes from baseline (BDC) and within-day AM-PM differences (pre vs post-EVA). Multiple comparisons adjustments were applied within each model to control Type 1: Dunnett corrections for baseline comparisons and simulation-based adjustment (ADJUST=SIMULATE) for AM-PM contrasts.

HIF-1α is normally present at very low concentrations or undetectable in normoxic serum due to rapid oxygen-dependent degradation. Because 63% of HIF-1α values were below the detection limit (<20 pg/mL), values were dichotomized as above detection limit (ADL) or not detected. A mixed-effects logistic regression including time category (Baseline; MD AM representing hypoxia; MD PM representing post-hyperoxia) as a fixed effect and a random subject-within-mission intercept was used to model detectability. Odds ratios with 95% confidence intervals are reported. Censored-data models (e.g., Tobit) were examined but rejected due to biased population-mean estimates in the presence of a high proportion of censored values.

Pearson correlations were used to evaluate associations between serum iron and previously published immune function markers (Crucian et al., 2025) at designated timepoints.

## Results

On average, the crews lost 1.6 ± 1.6% (mean ± SD) of their body weight by MD10. Only 3 of the 24 crewmembers maintained or had an increase in body weight by MD10.

### Responses during hypoxic phases

#### Metabolism

**Figure 1:**
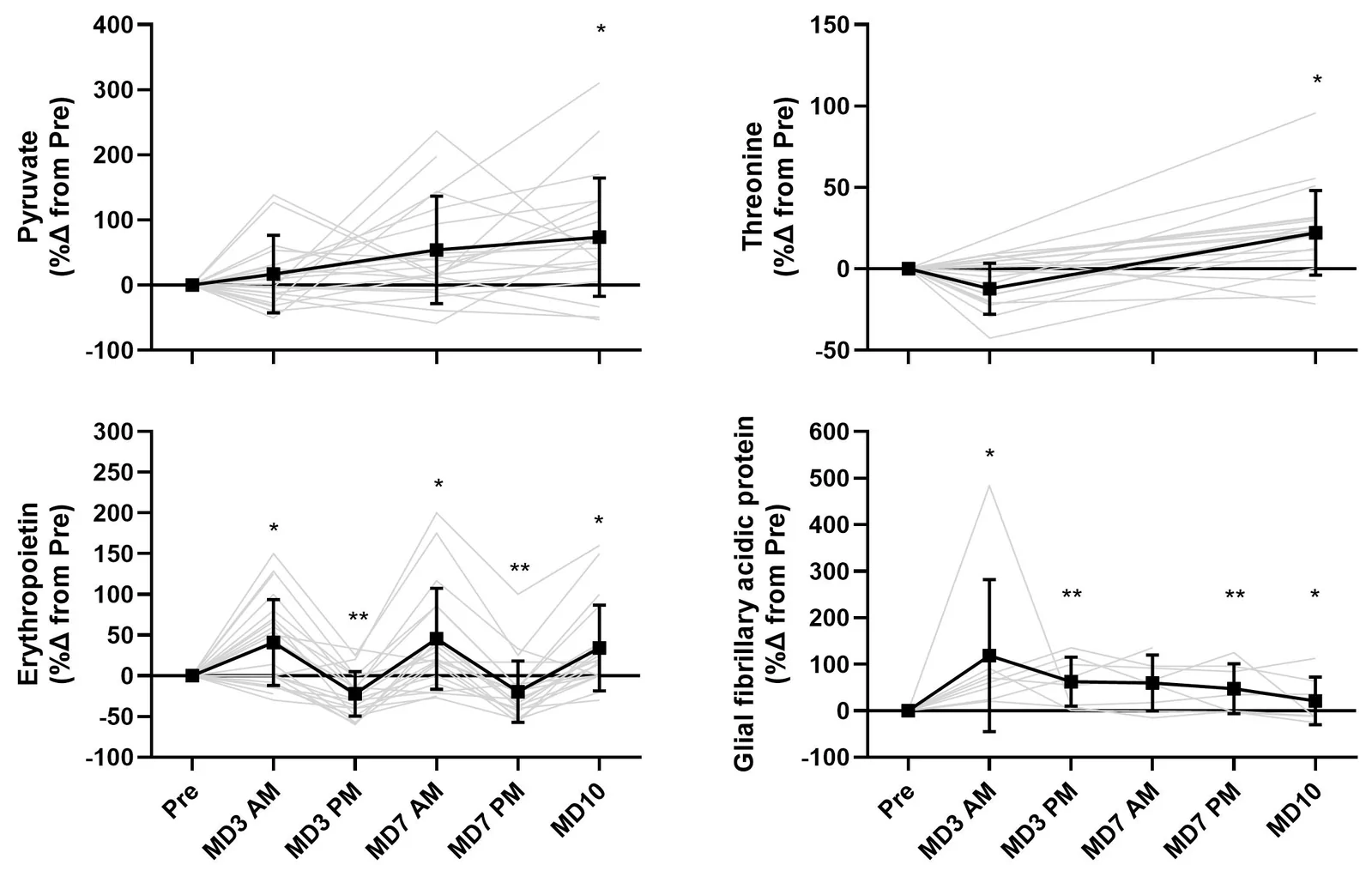
Percent change in pyruvate, threonine, erythropoietin, and glial fibrillary acidic protein during hypoxic and hyperoxic chamber conditions. Hyperoxic EVAs were conducted on MD3 and MD7 between the AM and PM blood collections. *Significant difference from baseline (p<0.05), **Significant difference from AM session (p<0.05).

The hypoxic environment in the chamber on non-EVA days resulted in transient changes in amino acid and glucose metabolism, as evidenced by changes in 2-aminoadipic acid, glutamine, leucine, malate, sarcosine, threonine, tyrosine and valine during compared to pre-mission (p<0.05, **Table 2**). These tended to be lower on MD3, but returned to baseline concentrations by the next collection at MD7. Other metabolites changed more persistently, as there was evidence of change not only at MD3, but at other time points as well (ß-alanine, carboxyethyllysine, methionine, phenylacetylglutamine, and proline), p<0.05. The percent change in threonine during the mission compared to baseline is presented in **Figure 1**, showing that while it is decreased early during the mission, by MD10 it is higher than baseline.

**Table 2:** Mean (± SEM) concentrations of amino acid and metabolism intermediates before and during exploration atmosphere testing.

Analyte	Function	Pre AM	MD3 AM	MD7 AM	MD10 AM
Amino acids and metabolites					
Fumarate (µmol/L)	TCA Cycle intermediate	1.5 ± 0.1	1.5 ± 0.1	1.5 ± 0.0	1.5 ± 0.0
Isocitrate (µmol/L)	TCA Cycle intermediate	3.4 ± 0.1	3.6 ± 0.1	3.3 ± 0.1	3.4 ± 0.1
Malate (µmol/L)	TCA Cycle intermediate	3.8 ± 0.2	**4.3 ± 0.2^a^**	4.2 ± 0.2	4.3 ± 0.3
α-Ketoglutaric acid (µmol/L)	TCA Cycle intermediate	6.4 ± 0.2	6.9 ± 0.3	6.7 ± 0.2	6.9 ± 0.23
α-Hydroxyglutaric acid (µmol/L)	Catabolite of a-ketoglutarate	0.6 ± 0.06	0.5 ± 0.03	0.5 ± 0.02	0.6 ± 0.0
Pyruvate (µmol/L)	Glycolysis end product	50 ± 5	58 ± 12	65 ± 8	**80 ± 9^a^**
Lactate (µmol/L)	Anaerobic glycolysis product	2000 ± 120	1800 ± 150	2100 ± 140	2300 ± 170
Glutamic acid (µmol/L)	Amino acid catabolism --> TCA cycle	38 ± 2	39 ± 3	35 ± 2	35 ± 2
Glutamine (µmol/L)	Amino acid catabolism --> TCA cycle	600 ± 17	**570 ± 17^a^**	580 ± 15	590 ± 16
Asparagine (µmol/L)	Nitrogen metabolism	70 ± 2	67 ± 3	72 ± 3	71 ± 2
Alanine (µmol/L)	Transamination	360 ± 15	350 ± 13	380 ± 14	370 ± 13
Proline (µmol/L)	Oxidation in mitochondria	170 ± 10	**140 ± 6^a^**	160 ± 7	**150 ± 7^a^**
Ornithine (µmol/L)	Urea cycle (mitochondrial matrix)	70 ± 3	**61 ± 3^a^**	67 ± 3	70 ± 3
Methionine (µmol/L)	One-carbon metabolism	31 ± 1	**28 ± 1^a^**	32 ± 1	**33 ± 1^a^**
Threonine (µmol/L)	One-carbon metabolism	150 ± 7	**140 ± 7^a^**	160 ± 10	**180 ± 9^a^**
Serine (µmol/L)	One-carbon metabolism	130 ± 5	130 ± 4	130 ± 4	130 ± 4
Glycine (µmol/L)	One-carbon metabolism	310 ± 17	290 ± 12	310 ± 11	310 ± 11
Sarcosine (µmol/L)	Glycine derivative, methyl donor	1.4 ± 0.1	**1.1 ± 0.1^a^**	1.3 ± 0.1	1.3 ± 0.1
Isoleucine (µmol/L)	Branched chain AA, ketogenesis	72 ± 3	65 ± 3	73 ± 3	79 ± 4
Leucine (µmol/L)	Branched chain AA, ketogenesis	140 ± 6	**130 ± 4^a^**	140 ± 4	140 ± 6
Valine (µmol/L)	Branched chain AA, ketogenesis	250 ± 7	**230 ± 6^a^**	250 ± 8	260 ± 10
β-Aminoisobutyrate (µmol/L)	Thymine catabolism --> TCA cycle	2.9 ± 0.2	3.1 ± 0.2	3.2 ± 0.2	**3.7 ± 0.2^a^**
Methylmalonic acid (µmol/L)	Functional marker of vitamin B12 status	0.15 ± 0.01	0.13 ± 0.01	0.13 ± 0.01	0.13 ± 0.01
Homocysteine (µmol/L)	One-carbon/methionine metabolism	8.1 ± 0.2	8.2 ± 0.2	8.2 ± 0.2	8.4 ± 0.3
Cystathionine (µmol/L)	Transsulfuration pathway	0.16 ± 0.01	**0.12 ± 0.01^a^**	0.14 ± 0.01	0.17 ± 0.01
Acetoacetate (µmol/L)	Ketone body	71 ± 13	53 ± 9.5	25 ± 10	44 ± 19
3-Hydroxybutyrate (µmol/L)	Ketone body	120 ± 83	82 ± 19	**29 ± 21^a^**	55 ± 30
3-Hydroxyisobutyrate (µmol/L)	Ketone metabolism	20 ± 2	17 ± 1	17 ± 1	19 ± 1
β-alanine (µmol/L)	Pyrimide degradation	3.8 ± 0.2	**3.2 ± 0.2^a^**	**4.4 ± 0.3^a^**	**4.4 ± 0.3^a^**
Cysteine (µmol/L)	Sulfur metabolism, antioxidant	280 ± 4	**290 ± 4^a^**	280 ± 5	280 ± 4
Lysine (µmol/L)	Essential AA, ketogenic	180 ± 7	170 ± 6	180 ± 6	190 ± 6
2-Aminoadipic acid (µmol/L)	Lysine catabolism --> Ketogenesis	0.79 ± 0.06	**0.67 ± 0.04^a^**	0.70 ± 0.05	0.73 ± 0.05
Kynurenine (µmol/L)	Tryptophan catabolism, NAD+ synthesis	1.6 ± 0.1	1.5 ± 0.1	1.5 ± 0.1	1.5 ± 0.1
Tryptophan (µmol/L)	Precursor for serotonin and NAD+	79 ± 3	75 ± 3	78 ± 3	78 ± 3
Phenylacetylglutamine (µmol/L)	Phenylalanine/Tyrosine pathway	19 ± 3	**31 ± 4^a^**	26 ± 2	**31 ± 3^a^**
Phenylalanine (µmol/L)	Phenylalanine/Tyrosine pathway	73 ± 2	70 ± 2	74 ± 2	75 ± 2
Tyrosine (µmol/L)	Phenylalanine/Tyrosine pathway	70 ± 1	**66 ± 2^a^**	70 ± 2	68 ± 2

Numbers in bold represent significant findings.^a^p<0.05 for vs Pre (BDC).

Endpoints measured at only AM (due to incomplete MD3 PM/MD7PM sampling) show only (a) indicators; PM contrasts were not evaluated.

Pyruvate was increased 60% after 11 days of living in the hypoxic conditions compared to baseline (**Figure 1**). ß-aminoisobutyrate was also increased 28% at the end of the mission (p<05).

Ketone 3-hydroxybutyrate was decreased on MD7 compared to baseline. Another ketone, acetoacetate, tended to be lower on that day but was not significantly changed during hypoxia exposure.

#### Oxidative stress/adaptive remodeling

There were several changes in markers of oxidative stress during the mission (**Table 3**). Superoxide dismutase increased at the end of the mission (MD7 and MD10). Total antioxidant capacity increased about 10% during exposure to hypoxia. Total lipid peroxides decreased during hypoxic phases of the study. Oxidized LDL was lower at the end of the mission compared to baseline.

**Table 3:** Mean (± SEM) concentrations of adaptive remodeling response before and during exploration atmosphere testing.

Analyte	Function	Pre AM	MD3 AM	MD3 PM	MD7 AM	MD7 PM	MD10 AM
Oxidative stress/damage and inflammation							
Catalase (nmol/m/mL)	Antioxidant	120 ± 23	190 ± 31	**140 ± 17^b^**	180 ± 29	**120 ± 16^b^**	180 ± 32
Superoxide dismutase (U/g Hgb)	Antioxidant	1100 ± 110	920 ± 45	840 ± 120	**1400 ± 76^a^**	**1100 ± 91^b^**	**1500 ± 30^a^**
Total Antioxidant Capacity (mmol/L)	Antioxidant status	1.0 ± 0.0	**1.1 ± 0.0^a^**	**1.2 ± 0.0^a^**	1.1 ± 0.0	**1.1 ± 0.0^a^**	1.0 ± 0.0
Total Lipid Peroxides (µmol/L)	Oxidative damage marker	0.54 ± 0.06	**0.35 ± 0.03^a^**	**0.35 ± 0.02^a^**	**0.34 ± 0.02^a^**	**0.33 ± 0.02^a^**	**0.32 ± 0.02^a^**
Glutathione, Reduced (µmol/L)	Antioxidant defense	640 ± 19	600 ± 30	**690 ± 19 ^ab^**	660 ± 20	**540 ± 32 ^ab^**	640 ± 25
Glutathione, Oxidized (µmol/L)	Oxidative stress marker	390 ± 18	**460 ± 18^a^**	**380 ± 18^b^**	380 ± 13	**420 ± 18^b^**	420 ± 30
Oxidized LDL (U/L)	Oxidative damage marker	51 ± 3	53 ± 3	50 ± 3	47 ± 2	**46 ± 2^a^**	**46 ± 2^a^**
HIF-1α (pg/mL)	Hypoxia signaling	120 ± 48	100 ± 42	120 ± 57	130 ± 58	110 ± 49	110 ± 44
HSP70 (ng/mL)	Stress response	2.3 ± 0.6	2.4 ± 0.7	**1.0 ± 0.4^b^**	1.8 ± 0.6	1.6 ± 0.4	3.6 ± 0.9
Glial fibrillary acidic protein (pg/mL)	Marker of blood-brain barrier integrity	76 ± 27	**150 ± 56^a^**	**79 ± 29 ^ab^**	71 ± 31	**79 ± 28^a^**	**60 ± 22^a^**
Glucose (mg/dL)	Primary energy source	90 ± 1	91 ± 1	90 ± 3	90 ± 1	88 ± 1	89 ± 1
Insulin (µU/mL)	Metabolic regulation	8.2 ± 0.8	8.5 ± 0.8	9.1 ± 2.5	**9.8 ± 0.9^a^**	**6.3 ± 0.7 ^ab^**	8.7 ± 0.8
hs CRP (mg/L)	Inflammation	2.0 ± 1.2	1.0 ± 0.4	1.0 ± 0.3	1.1 ± 0.3	1.1 ± 0.3	1.0 ± 0.3
Cortisol, Total (ng/mL)	Metabolic regulation	73 ± 6	**180 ± 3^a^**	38 ± 46	60 ± 73	**130 ± 11^a^**	100 ± 7
Carboxyethyllysine (nmol/L)	Advanced glycation end product	96 ± 9	120 ± 30		**140 ± 17^a^**		**260 ± 35^a^**
Carboxymethyllysine (nmol/L)	Advanced glycation end product	92 ± 9	100 ± 8		120 ± 12		98 ± 8
Urine N-telopeptide (nmol BCE/d)	Bone resorption	620 ± 4	**700 ± 5^a^**		**650 ± 6^a^**		**660 ± 5^a^**
Urine 8-hydroxy 2’-deoxyguanosine (µg/d)	Oxidative damage to DNA	12 ± 2	12 ± 2		12 ± 2		11 ± 2
Urine creatinine (mmol/d)	Kidney function	15 ± 1	13 ± 1		14 ± 1		15 ± 1
Urine volume (mL/d)	Hydration	2300 ± 210	**2700 ± 250^a^**		**3000 ± 270^a^**		**2900 ± 320^a^**
Urine pH		6.1 ± 0.1	6.1 ± 0.1		5.9 ± 0.1		6.2 ± 0.1

Numbers in bold indicate significant findings.^a^p<0.05 for vs baseline (Pre).

^b^p<0.05 for vs AM (same day).

Endpoints measured at only AM (due to incomplete MD3 PM/MD7PM sampling) show only (a) indicators; PM contrasts were not evaluated.

#### Stress/inflammation/bone

The number of individuals with HIF-1α above the detection limit was not changed during hypoxic periods (**Table 3**). No significant changes were found for glucose and hsCRP concentrations. GFAP (**Figure 1**) initially increased and then was decreased by the end of the study. Cortisol concentration increased early during the mission. Insulin increased after 7 days in the mildly hypoxic environment.

A urinary marker of bone resorption (N-telopeptide) increased during the mission on days 3, 7, and 10 compared to baseline (**Table 3**).

#### Hematology and vitamins

There were several changes in markers of iron metabolism, vitamin status, and related amino acid pathways. Erythropoietin consistently increased 22-34% after hypoxic exposure (MD3 AM, MD7 AM and MD10 AM, **Table 3**) (**Figure 1**). Soluble transferrin receptor levels increased 10% throughout the mission compared to baseline. Transferrin decreased later in the mission during hypoxic exposures. Vitamin C decreased upon hypoxic exposure.

Both red blood cell and serum folate increased with hypoxia. Cystathionine decreased on MD3. Homocysteine was unchanged during the mission.

### Responses to hyperoxic EVAs

#### Oxidative stress/adaptive remodeling

Catalase consistently decreased (p<0.05) after both hyperoxic EVAs (i.e., after both PM sessions). Superoxide dismutase increased at the end of the mission (MD7 PM and MD10). Total lipid peroxides decreased during the hyperoxic phases of the study. Reduced glutathione increased after the first hyperoxic EVA and decreased after the second one. Oxidized glutathione decreased after the first hyperoxic EVA and increased after the second one.

#### Stress/inflammation/bone

The number of individuals with HIF-1α above the detection limit was increased during the hyperoxic EVA periods. No significant changes were found for glucose and hsCRP concentrations during hyperoxic phases. HSP70 decreased after the first hyperoxic EVA, and tended to decrease after the second EVA as well. Meanwhile, GFAP (**Figure 1**) decreased during the first hyperoxic EVA and cortisol concentrations increased after the second hyperoxic EVA. Insulin decreased after the second hyperoxic EVA.

#### Hematology and vitamins

Erythropoietin consistently decreased 41-47% after both hyperoxic EVAs compared to the morning blood draw before the EVA (**Table 4**, **Figure 1**). Serum iron decreased 25% after each hyperoxic EVA exposure but returned to baseline on other days (**Table 4**). Total iron binding capacity (TIBC) increased after the hyperoxic EVAs. Vitamin C increased after each hyperoxic EVA exposure. Serum ferritin concentration decreased after the first hyperoxic EVA (**Table 4**).

**Table 4:** Mean (± SEM) concentrations of iron, hematology, and vitamin status before and during exploration atmosphere testing.

Analyte	Function	Pre AM	MD3 AM	MD3 PM	MD7 AM	MD7 PM	MD10 AM
Iron and Vitamin Status							
Erythropoietin (mU/mL)	Signaling to produce red blood cells	8 ± 1	**10 ± 1^a^**	**6 ± 0^ab^**	**11 ± 1^a^**	**6 ± 0^ab^**	**10 ± 1^a^**
Ferritin (ng/mL)	Tissue iron stores	48 ± 9	52 ± 10	**49 ± 9^b^**	47 ± 9	48 ± 9	43 ± 8
Transferrin (mg/dL)	Binds iron in circulation	270 ± 8	260 ± 6	270 ± 7	**250 ± 6^a^**	**250 ± 7^ab^**	**250 ± 7^a^**
Total iron binding capacity (µg/dL)	Available iron-binding sites on transferrin	340 ± 9	340 ± 8	**350 ± 9^b^**	330 ± 9	340 ± 9^b^	330 ± 9
Soluble Transferrin Receptors (mg/L)	Mediates iron uptake in erythroblasts	1.1 ± 0.1	**1.2 ± 0.1^a^**	**1.2 ± 0.1^a^**	**1.3 ± 0.1^a^**	**1.3 ± 0.1^a^**	**1.3 ± 0.1^a^**
Serum iron (µg/dL)	Total circulating iron	110 ± 8	100 ± 7	**82 ± 4^ab^**	97 ± 7	**79 ± 7^ab^**	120 ± 7.1
Vitamin C (µg/mL)	Antioxidant, enhances non-heme iron abs	11 ± 0.91	**9.3 ± 0.71^a^**	**10 ± 1^b^**	10 ± 1	**11 ± 1^b^**	11 ± 1.1
Red blood cell folate (nmol/L)	Folate status, required for erythropoiesis	400 ± 1	**500 ± 1^a^**	**540 ± 1^ab^**	**430 ± 1^a^**	**450 ± 1^ab^**	**540 ± 0^a^**
Serum folate (nmol/L)	Recent folate intake	29 ± 3	**35 ± 3^a^**	**38 ± 3^a^**	32 ± 4	**34 ± 3^a^**	30 ± 3

Numbers in bold represent significant findings.^a^p<0.05 for vs baseline (Pre).

^b^p<0.05 for vs AM (same day).

Red blood cell folate increased with hyperoxia.

#### Correlations

**Figure 2:**
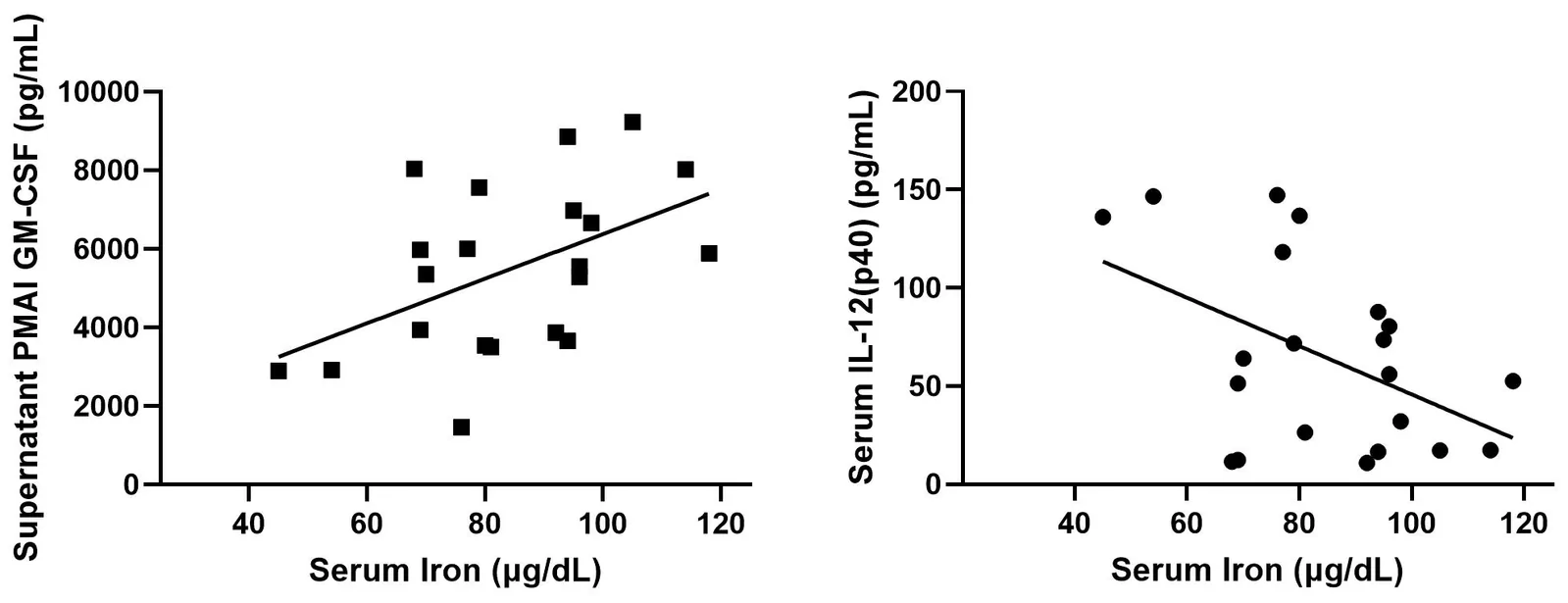
MD3 PM (after hyperoxic EVA exposure) relationship between serum iron and supernatant PMAI GM-CSF and serum IL-12(p40).

On MD3 PM, serum iron was negatively correlated with plasma cytokine IL-12p40 (**Figure 2**, Pearson r = -0.48, p=0.03) and positively correlated with GM-CSF production following leukocyte mitogenic stimulation (Pearson r=0.49, p=0.02). These results indicate that lower iron availability was associated with higher inflammatory cytokine concentrations and reduced leukocyte functional responses.

## Discussion

During periods of mild hypoxia, the physiological responses can be categorized as metabolic adaptation, redox/oxidative stress and adaptive response, and iron-erythropoiesis changes. Alternating from mild hypoxia to hyperoxic EVA stimulated several responses related to substrate utilization, transient changes in oxidative balance, and cyclic erythropoietic and iron handling.

### Response to hypoxia

The increase in cortisol during hypoxic conditions promotes catabolism and mobilization of glucose, and could also contribute to the observed changes in body weight, bone turnover, and immune-related responses. Pyruvate was significantly elevated above baseline by MD10. This is supportive of a shift in fuel homeostasis toward ATP production through the less efficient process of glycolysis instead of oxidative phosphorylation in the mitochondrial electron transport chain. It is also likely a protective mechanism because pyruvate can act as an antioxidant (H_2_O_2_ scavenger) and it protects mitochondria against oxidative stress (O'Donnell-Tormey et al., 1987; Desagher et al., 1997). The oxygen-sensing HIF-1α can increase glycolysis and allows pyruvate to accumulate as part of an antioxidant response (Semenza, 2011). Given the conditions were mildly hypoxic, HIF-1α activation may be due to a normobaric oxygen paradox response, where a relative decline in oxygen tension rather than absolute hypoxia, can trigger a hypoxia-like signal to stimulate erythropoiesis.

The decreases in ketones 3-hydroxybutyrate and acetoacetate during mild hypoxic exposure likely reflect a metabolic shift away from fat oxidation toward glycolysis. This is supported by the increase in pyruvate, stable glucose, elevated cortisol, and decreased circulating ketogenic amino acids early during the mission.

Mild hypoxic exposure decreased concentrations of several amino acids, possibly due to an increased amino acid utilization for responding to an increased stress response, increased metabolic use, protein turnover, and erythropoiesis. Threonine is an example of one amino acid that initially decreased early in the mission (MD3) and then increased by MD10. The biphasic response could be explained by changes in amino acid utilization or protein turnover, although the present dataset does not directly measure proteolysis and therefore, we cannot attribute the increase on MD10 to protein breakdown. The data support altered amino acid handling during hypoxia.

Erythropoiesis consumes iron and amino acids for protein synthesis, as well as one-carbon metabolism substrates such as glycine, methionine, and serine. An increase in erythropoiesis can increase bone marrow folate uptake into red blood cells, which increases red blood cell folate concentration (Antony et al., 1987). The increase in red blood cell folate observed during hypoxic conditions is supported by other studies showing that hypoxia can increase red blood cell folate likely by rapidly binding to hemoglobin when red blood cells are deoxygenated (Nelson et al., 1983), and this could potentially affect the circulating folate concentration. Increases in erythropoiesis can also increase transport and incorporation of folate into red blood cells (Koury et al., 2004).

The increase in oxidized glutathione and total antioxidant capacity could be a compensatory upregulation of antioxidant defense mechanisms. The decrease in vitamin C was likely because it is being consumed as one of the primary antioxidants. A decrease in lipid peroxides can occur when there is low oxygen that limits lipid oxidation.

Although several oxidative stress markers (increased total antioxidant capacity, fluctuations in reduced and oxidized glutathione) support activation of compensatory defense pathways, other classical indicators of oxidative damage did not consistently change and support increased oxidative stress overall. Lipid peroxides decreased across both hypoxic and hyperoxic phases, oxidized LDL was lower at the end of the mission, and urinary 8-OHdG did not change. The lack of a consistent pattern indicates that there may be transient shifts in redox balance or adaptation rather than an overall increase in oxidative injury.

Increased GFAP on MD3 of hypoxic exposure is supported by other studies in hypoxic conditions showing elevated GFAP, which is a biomarker for blood brain barrier dysfunction (Lumpkins et al., 2008). During hypoxia, a low level of astrocyte activation is expected, which can result in a mild blood-brain barrier stress (Lafuente et al., 2016). Interestingly, the GFAP response fluctuated during the periods of hyperoxia and hypoxic exposures, suggesting there may be an acute and chronic adaptive response to altered partial pressure of oxygen.

### Responses to hyperoxia

Hyperoxic EVAs triggered a distinct set of metabolic and oxidative responses. Decreases in erythropoietin following EVAs and increases in TIBC and soluble transferrin receptor concentrations are consistent with reduced erythropoietic signaling under high oxygen tension.

Glutathione synthesis increased and oxidized glutathione decreased with the first hyperoxic exposure but increased with the second one, indicating there could be inefficient recycling of glutathione. The decrease in catalase with hyperoxic exposure may be due to its inactivation in hyperoxic oxygen environments (Escobar et al., 1996). The increase in vitamin C concentration with hyperoxic exposure fits with the other findings of increased reduced glutathione and decreased oxidized glutathione, and this is an indication of enhanced antioxidant recycling.

The increase in urinary N-telopeptide during the mission indicates bone resorption was increased. The increase in cortisol may explain that response, as cortisol is a potent stimulator of bone resorption due to its role in releasing amino acids for use as an energy source through gluconeogenesis (Mathis et al., 2013).

Hypoxia is also known to upregulate, or sensitize, immune cell functional responses. We previously reported that for the present subjects, leukocyte function as defined by *in-vitro* mitogenic stimulation of leukocytes resulting in increased supernatant cytokine concentrations, was elevated post EVA (Crucian et al., 2025).

In addition to the core metabolic and adaptive changes, several patterns in the dataset suggest additional mechanisms that could contribute to the adaptive response during this unique environment with cycling between hypoxia and hyperoxia. The dissociation between increased pyruvate by the end of the mission and relatively stable lactate may reflect altered redox balance and preferential routing of pyruvate toward antioxidant or other non-lactate producing pathways. The gradual increase in circulating advanced glycation end products indicates there is some cumulative oxidative stress across the cycles of oxygen availability. Finally, the intermittent detectability of HIF-1α suggests there may be a transient stabilization rather than a classical hypoxic activation.

### Iron-erythropoiesis axis

The iron-erythropoiesis axis is a central integrating mechanism that is involved in coordinating physiological responses observed in the repeated hypoxic-hyperoxic transitions. Mild hypoxia consistently stimulated erythropoietin production and increased soluble transferrin receptor and red blood cell folate concentrations, reflecting an increased drive for erythropoiesis and increased utilization of one-carbon substrates. Each hyperoxic EVA produced reductions in serum iron and erythropoietin along with increases in TIBC, suggesting transient iron sequestration or redistribution in response to higher oxygen exposure. These fluctuations in iron were aligned with changes in antioxidant capacity, vitamin C status, and immune responses.

Iron is essential for optimal immune functioning, influencing both innate and adaptive immunity, promoting efficient immune cell proliferation, activation, and differentiation. Appropriate iron levels support T-cell, B-cell, and macrophage function, while suboptimal concentrations can severely compromise immune responses and increase susceptibility to infection. Serum iron decreased markedly after each EVA, consistent with transient redistribution or sequestration in response to hyperoxic stress.

Here, we note a positive correlation between serum iron and GM-CSF production following leukocyte mitogenic stimulation *in vitro* (**Figure 2**), and the negative correlation with IL-12 (p40), suggest that reduced iron availability may blunt immune activation and increase inflammation. This supports the notion that proper nutritional status is a prerequisite for maintaining adequate immune function and reducing specific clinical risks during deep space missions. These missions will uniquely, in the history of spaceflight, consist of cycling between hypoxic and hyperoxic conditions. IL-12 is produced by several cell types, but primarily by antigen presenting innate leukocytes such as monocytes/macrophages. We suggest this finding also fits our immune-nutrition-atmosphere hypothesis, as adequate iron reserves results in increase leukocyte function and efficiency, which may therefore reduce ‘baseline/systemic’ subject inflammation.

### Limitations

Interpretation of some metabolic endpoints is limited by the lack of PM amino acid and glucose measurements at MD3 and MD7, due to incomplete sampling and budget constraints. The relatively small sample size and short mission duration also limit generalizability. Additionally, biomarkers vary in their response times to hypoxia and hyperoxia; thus, the alternating exposure schedule may have obscured some transient effects or blended acute and cumulative responses. Furthermore, it is important to note that the blood samples were collected in the chamber, but were transferred to the lab for analyses within an hour. That duration may be long enough to degrade some analytes that may be labile (including HIF-1α).

### Implications for exploration missions

Despite these limitations, the study provides important initial insight showing that alternating exposure to mildly hypoxic conditions and hyperoxic EVAs produced metabolic adaptations, redox remodeling without consistent oxidative damage, and changes in the iron-erythropoiesis axis. The findings underscore the significance of spacecraft and spacesuit atmospheric design and highlight the need to refine nutritional and operational countermeasures that support crew health, performance, and resilience during deep-space missions. A better understanding of nutrient requirements for exploration class missions and environments will be critical for ensuring mission success.

## Data Availability

The datasets are governed by NASA’s Human Research Program and fall under restrictions outlined in the NASA IRB approval and subject consent structure. Access to the datasets requires controlled review and approval through NASA’s Life Sciences Portal (https://www.nasa.gov/hrp/nlsp/). Requests for access to the restricted data should be directed to the Life Sciences Portal under “Request Data”, and both de-identified and possibly attributable (identifiable) data may be available for peer-reviewed research studies following a thorough review and approval process and NASA IRB approval.
